# Efficient Saturable Absorber Based on Ferromagnetic Insulator Cr_2_Ge_2_Te_6_ in Er-Doped Mode-Locked Fiber Laser

**DOI:** 10.3390/nano12050751

**Published:** 2022-02-23

**Authors:** Ruyi Sun, Linguang Guo, Xinxin Shang, Huanian Zhang, Qingyang Yue

**Affiliations:** 1 Shandong Provincial Key Laboratory of Optics and Photonic Devices, School of Physics and Electronics, Shandong Normal University, Jinan 250358, China; ruyi_sun1@163.com (R.S.); linguang_guo@163.com (L.G.); xinxin_shang10@163.com (X.S.); 2 School of Physics and Optoelectronic Engineering, Shandong University of Technology, Zibo 255049, China; 3 Shandong Ruixing Single Mode Laser Technology Co., Ltd., Zibo 255049, China

**Keywords:** Cr_2_Ge_2_Te_6_, saturable absorber, mode locked, Er-doped fiber laser

## Abstract

A ferromagnetic insulator Cr_2_Ge_2_Te_6_ as a saturable absorber in an Er-doped fiber laser (EDFL) was demonstrated. In this work, a CGT-PVA composite film was successfully fabricated using the liquid-phase exfoliation method and employed in an EDFL. The modulation depth and saturation intensity of the SA are 4.26% and 89.40 MW/cm^2^, respectively. Stable pulses with a minimum pulse width of 978.5 fs when the repetition rate was 3.25 MHz were recorded experimentally. Furthermore, stable solitons still need to be obtained when the pulse energy in the cavity is as high as 11.6 nJ. The results fully suggest that CGT has outstanding nonlinear absorption properties, which may have broad potential applications in ultrafast photons.

## 1. Introduction

Two-dimensional (2D) materials have exhibited a lot of interesting optical properties when their thickness is close to a few atomic layers. Their excellent optical performances mean that they are widely applied in photo-detection [1,2], biological sensing [3,4], and optical modulation [5,6]. Diverse SAs based on 2D materials were applied to modulate ultra-fast lasers. The history of two-dimensional materials as SA can be traced back to applications of graphene. In 2009, graphene was applied as a saturable absorber (SA) in an EDFL, and a 756 fs ultrashort pulse was obtained [7]. Since then, the studies of SA based on 2D materials have prevailed. However, the absorption of monolayer graphene is 2.3%, limiting its application and development in the field of optoelectronics [8]. Afterwards, transition metal disulfides (TMDs) [9,10,11,12,13,14], topological insulators [15,16,17,18,19,20], black phosphorus [21,22,23,24] and other 2D layered materials [25,26,27,28,29,30] with excellent saturable absorption have also been adopted in ultrafast photonics. These two-dimensional materials have a similar layered structure, in that the layers are connected through van der Waals forces, which provides the possibility to obtain single or multiple layers from the bulk materials. Benefiting from their smaller energy band gap, they can be operated at different wavelengths. The chemical formula of TMDs is MX_2_ (in which M: Mo, W, Nb, etc; X: S, Se, Te, etc.), and they may behave as insulating, semiconducting, or metallic substances [31,32]. Sue to the existence of weak van der Waals forces between layers, it is possible to exfoliate few layers from the bulk [33]. Many studies examining ultrafast fiber lasers based on 2D TMDs as SAs have been reported [34,35,36,37]. However, TMDs devices are not suited for the mid-infrared region, since the intrinsic energy bandgap of TMDs is limited from 1 to 2 eV [38]. BP has attracted great interest in both potential applications and academic research recently, benefiting from its remarkable characteristics. Differing from graphene and TMDs, the puckered structure of BP leads to a high degree of anisotropy of light absorption and photoluminescence [39], and the band gap of BP bridges the gap between graphene and TMDs. However, the weak thermal stability of the BP has limited its further development and so on. Therefore, the investigation of SA materials with large saturable absorption and a high damage threshold continues to be required [40,41,42,43,44].

Ferromagnets such as Cr_2_Ge_2_Te_6_ (CGT) belong to the R3 space group. The quasi-hexagonal crystal structure of CGT consists of a three-layer-stacked structure of ABC sequence [45]. The microstructure determines its special energy band. The theoretical band gap value (Eg) of CGT is about 0.74 eV [46,47]. CGT has been widely investigated due to its interesting lattice and band structures. For example, Ji et al. used CGT as a model system for the growth of Bi_2_Se_3_ and obtained large continuous thin films of Bi_2_Se_3_ [48]. In 2018, Xie et al. reported that 2D CGT has excellent ultra-sensitive photoresponses, which can detect weak light at incident power as low as 0.04 pW [49]. In addition, the magneto-optical effects [50] and magneto-elastic coupling phenomenon [51] have been widely researched. Compared with the mentioned 2D materials, the CGT exhibited a similar layered hexagonal structure and a suitable bandgap value. The modulator based on CGT is expected to be applied to ultrafast photonics. However, the research of nonlinear absorption properties of CGT is relatively deficient.

In our work, CGT-SA was successfully prepared and used in a mode-locked EDFL. The saturation intensity and modulation depth of CGT-PVA SA are 89.40 MW/cm^2^ and 4.26%, respectively. A stable mode-locked EDF laser operating at 1558.9 nm is demonstrated using CGT-SA. The narrowest pulse width is 978.5 fs. The maximum output power was 4.207 mW and the corresponding single pulse energy was 1.29 nJ when the fundamental repetition frequency was 3.25 MHz. In addition, the CGT-based EDFL can still work stably when the intracavity single pulse energy is as high as 11.6 nJ. The excellent performance of our EDFL indicates that CGT is a desirable candidate in ultrafast modulation.

## 2. Preparation and Characterization of the CGT SA

### 2.1. Preparation of CGT-Based SA

Various structures of SA, including the D-shaped fiber, the sandwich structure and so on, have been employed in mode-locked EDFL. The D-shaped fiber and the tapered fiber adopted indirect evanescent field coupling, which provides an interaction between material and laser. The evanescent field decreases exponentially with distance. Compared with the evanescent field, the direct interaction between the light and SA by inserting the SA between two optical fiber connectors is more effective for achieving mode-locked operation.

Due to the van der Waals force between different layers, the liquid-phase exfoliation is a more effective method to obtain CGT nanosheets. Firstly, 0.15 g of CGT powder was mixed with 15 mL of alcohol (30%) in a clear bottle, and the mixture was put in an ultrasonic cleaner for 10 h. In this step, the CGT was sufficiently dispersed via ultrasonic waves. Secondly, the 5 mL CGT dispersion and 5 mL 5 wτ.% PVA solution were mixed through a 4 h ultrasonic process. Finally, the 50 µL CGT-PVA dispersion solution was drop-coated on a glass substrate and placed into an oven for 12 h at 30 °C. According to the above steps, the CGT-PVA film was obtained. Finally, we cut off a 1 × 1 mm^2^ film and put it on the end of an optical fiber jumper, and the SA based on CGT nanosheet was fabricated successfully.

### 2.2. Characterization of CGT SA

**Figure 1:**
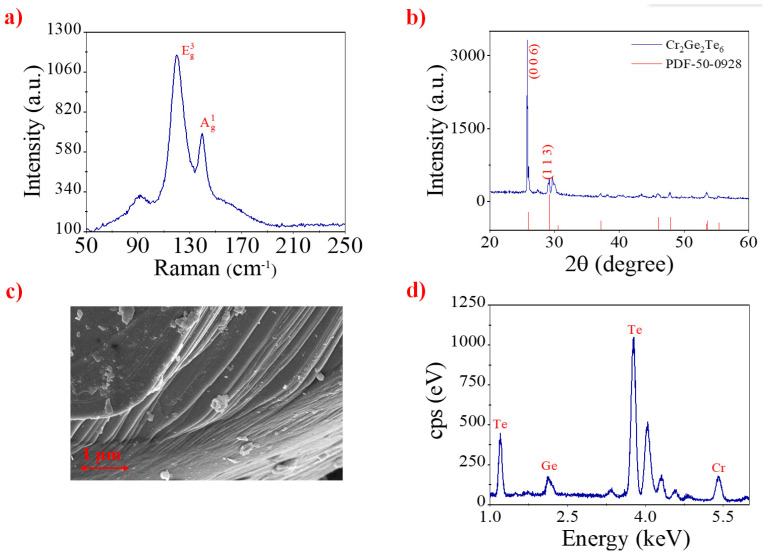
(**a**) Raman spectrum of CGT nanosheets, (**b**) XRD analysis for CGT nanosheets, (**c**) SEM image of CGT nanosheets, and (**d**) corresponding EDS spectrum of CGT nanosheets.

The Raman spectrum of CGT powder was recorded, and is presented in Figure 1a. Obviously, it includes two main peaks in 120 and 139 cm^−1^, corresponding to the typical Eg^3^ and Ag^1^ mode of CGT [46]. This indicates that the CGT has a high purity. The X-ray diffraction (XRD) (D8 Advance, Bruker, Billerica, MA, USA) analysis is presented in Figure 1b, and high diffraction peaks at (006) and (113) mean that CGT has a well-layered structure and high crystallinity. The surface morphology and layered structure were recorded with a scanning electron microscope (SEM) (Sigma 500, ZEISS, Oberkochen, Germany). Figure 1c shows the obvious layered structure and distinct boundaries between different layers. The interaction between layers is the van der Waals force, and the intensity is weak; therefore, the CGT nanosheets with few layers could be extracted from the bulk though ultrasonic operation. Furthermore, the bandgap was related with the thickness of the material, and the different bandgap values could be acquired for different thicknesses. In order to analyze the composition of our sample, Figure 1d shows the energy dispersive spectrum (EDS) (QUANTAX EDS, Bruker, Germany), and the atomic ratio is nearly 1:1:3 (Cr:Ge:Te), which corresponds with the chemical formula of CGT. Combined with the above tests, it is obvious that the purity of CGT is relatively high.

**Figure 2:**
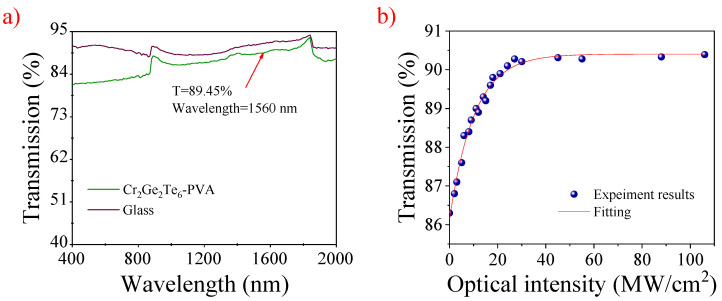
(**a**) Linear transmission versus wavelength of the CGT-PVA film, (**b**) the nonlinear absorption property of the CGT-PVA film.

In Figure 2a, the transmittance of the film is recorded by a UV/Vis/NIR spectrophotometer (U-4100, Hitachi, Tokyo, Japan). It shows a large absorption range from 400 to 2000 nm. The transmission is about 89.45% at 1560 nm. In addition, the double-balanced detection system was used to record the saturable absorption of SA. The system includes an ultra-short pulsed laser source (central wavelength: 1580 nm, repetition rate: 33.6 MHz, pulse duration: 560 fs), an adjustable attenuator, a 1:1 fiber coupler and a power meter. By adjusting the attenuator, the transmitted power increased and tended to be constant when incident optical power was further increased. Obviously, the optical transmittance became saturated. In Figure 2b, the experimental data were fitted by the following equation [52,53]:

$$T\left(I\right)=1-T_{ns}-\Delta \cdot exp\left(-\frac{I}{I_{sat}}\right)$$

where *T(I)* and *T_ns_* represent the transmission rate and the nonsaturable loss, respectively. *I* and *I_sat_* are the input pulse energy and the saturation intensity, respectively, and Δ is the modulation depth.

It can be calculated that the modulation depth and the saturation intensity are 4.26% and 89.40 MW/cm^2^, respectively.

## 3. Experimental Results and Discussion

**Figure 3:**
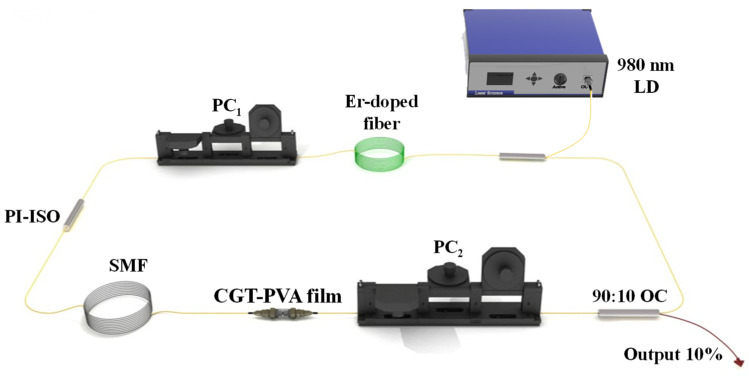
The experimental setup of the mode-locked laser.

The experimental construction of the EDFL is presented in Figure 3. The pump source is a 980 nm laser diode (LD, Shandong Ruixing Single Mode Laser Technology Co. LTD, Zibo, China) and the maximum output power is 1.3 W. A 980/1550 wave division multiplexer (WDM, Jinan Jingjiang photoelectric technology co. LTD, Jinan, China) transmits the pump power into the cavity. About 10% of the laser energies were output from a 10:90 optical coupler. The polarization state was adjusted by two polarization controllers (PCs, Jinan Jingjiang photoelectric technology co. LTD, Jinan, China). A polarization-independent isolator (PI-ISO, Jinan Jingjiang photoelectric technology co. LTD, Jinan, China) provides a unidirectional transmission of the light in the ring cavity. A length of 39.7 cm Er-110 (dispersion value: −46 ps/(km·nm)) was employed as a gain medium in the laser cavity. In addition, experimental data were recorded by other devices, including a digital oscilloscope (Wavesurfer 3054, Teledyne LeCroy, Thousand Oaks, CA, USA), a power meter (PM100D-S122C, Thorlabs, New Jersey, USA), an optical spectrum analyzer (AQ6317B, Yokogawa, Yokogawa, Tokyo, Japan), a photo-detector (PD-03, Shandong Ruixing Single Mode Laser Technology Co. LTD, Zibo, China) and a spectrum analyzer (R&S FPC1000, Jena, Germany).

Initially, no pulse generated through adjusting the PCs before the CGT-SA was inserted into the ring fiber cavity. Then, the SA was inserted into the cavity, and a mode-locked pulse was obtained by rotating the PCs and changing pump power. These results indicate that the CGT-SA was a necessary component in the EDFL.

**Figure 4:**
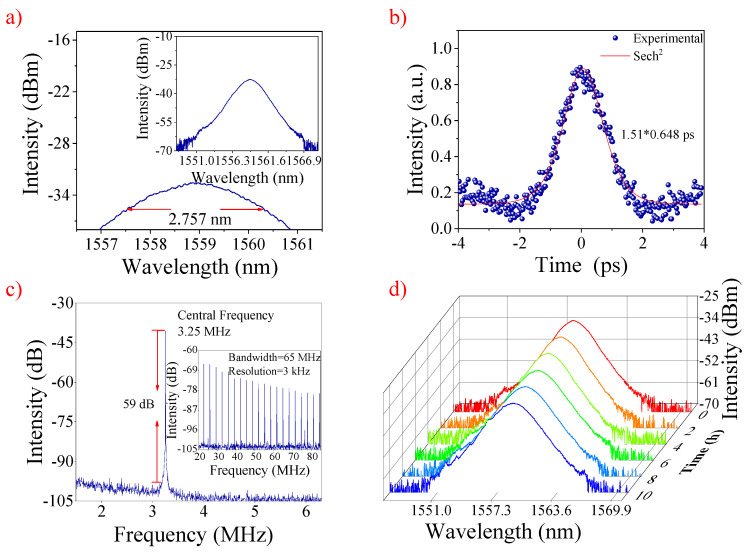
(**a**) Typical optical spectrum, (**b**) autocorrelation trace and sech^2^ fitting of the output pulse, (**c**) the RF optical spectrum at the fundamental frequency of 3.25 MHz. Insert: the broadband RF output spectrum. (**d**) The spectral states versus time.

The optical spectrum is presented in Figure 4a under the pump power of 252 mW. The central wavelength and 3 dB bandwidth are 1558.9 nm and 2.757 nm, respectively. The photon energies were calculated to be 0.796 eV, which is higher than the 0.74 eV bandgap value of CGT. Therefore, the CGT can be used as a wideband SA. An autocorrelator was used to record the real width of pulses. Figure 4b shows the auto-correlation of the output mode-locked pulse, and the width was about 978.5 fs.

In this work, the τ_pulse_, λc, and Δλ were 978.5 fs, 1558.9 nm, and 2.757 nm, respectively. Therefore, the time-bandwidth product (TBP) was calculated to be 0.333. Compared with the theoretical limited values (0.315), higher TBP values mean that the mode-locked pulses were weakly chirped. Figure 4c shows the radio frequency (RF) spectrum of the single pulse, for which the signal-to-noise ratio was 59 dB under the fundamental repetition rate of 3.25 MHz. Meanwhile, the optical spectrum of the laser under the pump power of 252 mW was recorded at intervals of 2 h. Figure 4d shows that the central wavelengths of the optical spectrum are 1558.9 ± 0.3 nm, and the change in the 3 dB bandwidth was less than 0.4 nm. Thus, the long-term stability of the laser is considerably good [54,55].

Considering the limitations of the soliton area theorem, the bright solitons can reach a near zero negative dispersion area when the balance between the total gain and loss is reached. The total length of the laser cavity is 63 m, including 62.8 m SMF (dispersion value: 17 ps/km·nm) and 0.397 m EDF (dispersion value: −46 ps/km·nm) in the cavity. The net dispersion of cavity was estimated to be −1.35 ps^2^. In addition, the output power is 120 µW at minimum pump power and the corresponding pulse energy is 0.037 nJ, which is lower than the limited values (0.1 nJ) of conventional solitons.

**Figure 5:**
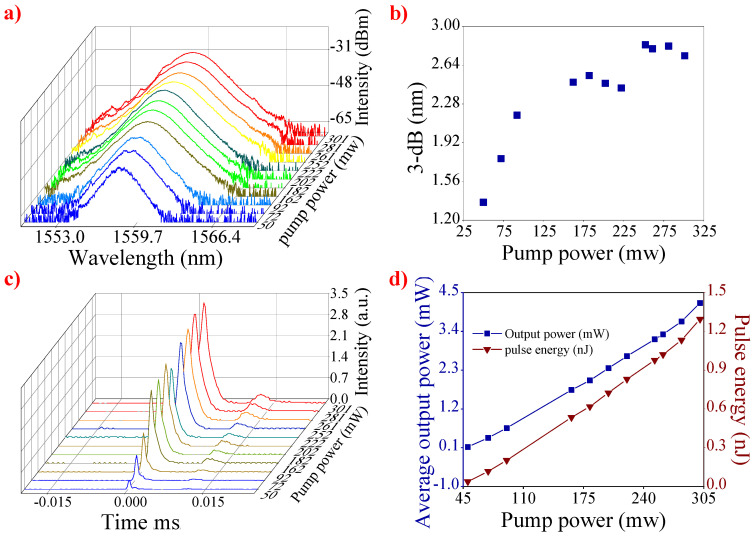
(**a**) Spectral evolution versus pump power, (**b**) 3 dB bandwidth value of the optical spectrum at different pump powers, (**c**) the pulse evolution versus pump power, and (**d**) the output power and pulse energy versus pump power.

The optical spectrum at different pump powers is presented in Figure 5a. With the rise in pump power, the shape of the spectrum is a smooth curve, and the central wavelength does not shift, which indicates that the mode-locked operation operates stably under pump power from 50 to 301 mW. As depicted in Figure 5b, the 3 dB bandwidth has a slight broadening, which is mainly caused by the increase in the pump power. Figure 5c shows the shape of a single pulse at different pump powers. The intensity of the pulse rises slightly as the pump power increases. This suggests that the mode-locked laser has outstanding stability. In Figure 5d, it is obvious that the output power and single pulse energy rise with the increase in the pump power, and the maximum output power is 4.207 mW, corresponding to the single pulse energy of 1.29 nJ. Because the 10% energy is output through the coupler, the power in the cavity was calculated to be 37.86 mW (corresponding to 11.6 nJ single pulse energy). When the pump power further increased, we were not able to achieve stable mode-locked operation. However, the pulse could be obtained when the pump power decreased under 301 mW. Clearly, the CGT-PVA SA has a high thermal damage threshold.

## 4. Conclusions

In conclusion, FI-CGT SA as a modulator was applied in an EDF mode-locked laser. A stable 978.5 fs pulse at a central wavelength of 1558.9 nm was obtained. The saturable intensity and the modulation depth of the SA based on CGT are 89.40 MW/cm^2^ and 4.26%. The single pulse energy is 1.29 nJ under a repetition rate of 3.25 MHz. Our experiment demonstrated that CGT has good nonlinear absorption characteristics and will have promising applications for ultra-photonics.

## Data Availability

The data presented in this study are available on request from the authors.
